# Relationship between body composition and physical fitness of primary school learners from a predominantly rural province in South Africa

**DOI:** 10.4102/phcfm.v14i1.3517

**Published:** 2022-09-07

**Authors:** Howard Gomwe, Eunice Seekoe, Philemon Lyoka, Chioneso Marange, Denford Mafa

**Affiliations:** 1 Department of Teaching, Learning and Community Engagement, Sefako Makgatho Health Sciences University, Pretoria, South Africa; 2 Department of Human Movement Sciences, Faculty of Health Sciences, University of Fort Hare, East London, South Africa; 3 Department of Statistics, Faculty of Science and Agriculture, University of Fort Hare, East London, South Africa; 4 Department of Social Work, Faculty of Social Sciences and Humanities, University of Fort Hare, East London, South Africa

**Keywords:** BMI, body composition, children, physical fitness, primary school

## Abstract

**Background:**

There is a lack of literature regarding the relationship that exists between body composition and physical fitness amongst primary school learners in South Africa. For the sake of public health purposes, it is important to investigate how body composition relates to physical fitness amongst primary school learners in the Eastern Cape province of South Africa.

**Aim:**

The aim of this study was to examine the relationship between body composition and physical fitness amongst South African primary school children.

**Setting:**

The study was conducted on a cohort of primary school learners in the Eastern Cape province, which is a predominantly rural province in South Africa.

**Methods:**

A school-based cross-sectional survey was conducted amongst 870 primary schoolchildren aged 9–14 years. Body composition and physical fitness measurements were measured and recorded using standardised measurement scales.

**Results:**

Of the 870 participants, 40.34% (*n* = 351) were boys and 59.66% (*n* = 519) were girls. The mean age of the participants was 11.04 ± 1.50 years. Boys had a significantly (*p* = 0.002) higher mean age (11.24 ±1.51 years) as compared to girls (10.91 ± 1.48 years). The results of the non-parametric Spearman’s rho correlation coefficients revealed several significant and negative relationships between physical fitness and body composition measurements, which were stronger in girls than in boys.

**Conclusion:**

The findings call for public health authorities and other relevant policymakers to initiate the development and implementation of policies and interventions targeted at encouraging physical activity participation and healthy lifestyle amongst primary school learners in South Africa, especially amongst girls.

**Contribution:**

The study findings supports a relatively rich literature which suggests that girls are more flexible than boys and that negative relationships between body composition measurements and physical fitness characteristics exists, which are stronger in girls than in boys.

## Introduction

One of the determinants of good health amongst adolescents is body composition.^1,2^ Body composition comprises muscle, fat, tissue and bone.^3^ There is a wide publication of literature on the relationship between physical fitness and body composition amongst learners in high-income countries.^4,5,6,7^ However, this has not been the case in middle-income countries, where there is scanty literature related to the phenomenon under study. In South Africa, although there has been a high prevalence of overweight and obesity amongst children,^8,9,10,11^ there are few research studies regarding the relationship between physical fitness and body composition amongst primary school learners.

Physical fitness is defined as the ability of the body to function successfully and proficiently, being healthy and being able to resist disease, enjoy leisure and to adjust to different conditions.^12,13^ There are two basic types of physical fitness. One is health-related, which means the general well-being of the body, and the other is skill-related, which is the ability to perform some aspects of sports.^12,14,15,16,17^ Health-based physical fitness comprises cardiovascular fitness, flexibility, body composition, muscular endurance and strength, whilst skill-based aspects include balance, agility, speed reaction time, power and coordination.^18^

According to some studies,^19,20^ both boys and girls with high body mass index (BMI) performed poorly as compared to those with normal BMI in exercises such as push-ups, sit and reach, shuttle run and sit-ups. Similar findings have confirmed a negative relationship between physical fitness and body fat.^21,22^ Learners with high BMI struggle to breathe when it comes to strenuous exercises because of limited oxygen supply in their bodies.^23,24,25^ Generally, it is thought that a low body fat percentage (FATp) is desirable to thrive in sports, whilst a high FATp is not desirable at all.^26^ An assessment of the relationship between physical fitness and composition of the body in school learners in middle-income countries like South Africa is not common. Body composition provides an estimate of children’s weight that is composed of fat tissue, contrary to lean body mass.^27^ Maintaining good health is very important in preventing obesity and overweight with its related non-communicable diseases.^28^

High BMI is associated with obesity and overweight, leading to increased risk of chronic diseases.^29^ This is usually because of unhealthy diet and physical inactivity.^30^ Similarly, a research study conducted on well-fed urban learners in South Africa also reported relationships between physical fitness and body composition that were contrasting.^31^ In South Africa, there is no clear research evidence to suggest whether the relationship between physical fitness and body composition of undernourished rural learners is the same as that of urban learners. Overweight is more common in urban learners,^32^ and the prevalence of underweight is much more common in township school learners.^33^ To date, in South Africa, there are few studies that have investigated the relationship between physical fitness and body composition. However, it can be noted that of the existing studies, the majority are on underweight learners.^34,35^

Physical fitness is one of the most vital targets in reducing childhood obesity and overweight through physical activity habits and a healthy lifestyle.^36,37^ It is important to increase physical fitness in order to improve the well-being of children and prevent diseases. It is also important to understand the differences in body composition and physical fitness of learners for the purpose of health promotion. Moreover, there are few research studies comparing the differences in physical fitness and body composition of rural learners in South Africa. Therefore, this study seeks to determine the relationship between physical fitness and the body composition of rural-based school learners in South Africa.

## Research methods and design

### Study design

The study adopted a quantitative approach and made use of a cross-sectional study to assess the relationship between body composition and physical fitness of primary school learners in the Eastern Cape province of South Africa.

### Study setting

The research study was conducted in three purposively selected municipalities within the Eastern Cape province of South Africa. These include Buffalo City Metropolitan, Oliver Tambo and Chris Hani municipalities, which were selected for the purposes of comparing the diverse contexts that exist in the targeted geographical space. The study setting is predominantly rural, but the three municipalities represent both urban and rural communities.

### Study population and sampling strategy

A random selection was based on a list of quintile 1, 2 and 3 schools which was provided by the district education departments of each selected municipality. The quintile ranking is a system that the South African Department of Education uses to divide public schools for purposes of allocation of financial resources, where quintile 1 has schools which are the poorest, whilst in quintile 5 there are schools which are the least poor. This study only considered schools that are poor and non-fee paying. Using a computer-generated programme, 18 primary schools were randomly selected. They included six schools (urban schools) from Amathole municipality, six schools (rural schools) from Oliver Tambo municipality and six schools (rural schools) from Chris Hani municipality. Lastly, class registers were used to randomly select a 10th of the population from each selected school. A total of 870 (411 from urban schools and 459 from rural schools) participants were recruited and participated in the study.

### Data collection

For the participating learners, anthropometric and physical fitness measurements were recorded using standardised measurement scales as described below.

### Anthropometric measurements

The anthropometric measurements were measured as follows:

- *Weight:* The weight was measured in kg using a scale.
- *Height:* The height was measured in cm using a stadiometer.
- *Body mass index (BMI)*: This was calculated using weight and height.^38^
- *Triceps and subscapular:* These were calculated using a formula.^39^
- *Waist circumference (WC):* WC was measured (in cm) and a tape measure was used. The measurement was taken at the narrowest area, that is, below the rib cage and above the umbilicus cord, as viewed from the front.
- *Waist-to-hip ratio (WHR):* To determine WHR, WC was measured (in cm) and then divided by measurements of hip-circumference (HC).
- *Hip gluteal:* Without indenting the soft skin tissue of the participant, a tape measure was used to measure hip gluteal.

### Measurement of physical fitness

The EUROFIT protocol was used in the process of physical fitness measurements.^40^ The physical fitness tests involved sit-ups (muscular endurance), sit and reach tests (flexibility), push-ups (abdominal strength) and a 20 m shuttle run (cardiorespiratory endurance). The sit and reach tests measure the flexibility of the lumbar and hamstring muscles and are now widely used as a reliable test of flexibility in children.^41^ Strength and endurance were measured using push-ups. According to the American College of Sports Medicine,^42^ muscular endurance is the ability of body muscles to repeat muscle contractions over a long period without fatigue. It is a good predictor of muscle strength in physical activity. Abdominal muscle endurance was measured using sit-ups. The learners started by lying on the back with the knees at a right angle, whilst another learner held the ankles of the participant, tightly pressing them downwards. The girls and boys were tested in separate classrooms. The numbers of sit-ups scored within 1 min were recorded on the scoring sheet.^43^ Cardiorespiratory fitness was assessed using a validated maximal multistage 20 m shuttle-run test. This test predicts maximal aerobic capacity and could be used as a measure of aerobic fitness in school learners. The test usually comprises 23 levels where each level is a sequence of 20 m shuttle runs. Each level takes at least 1 min and the time between the recorded ‘beeps’ lessens for each new level. School learners were familiarised with the procedure first. The results were entered as number of laps per level taken to complete the 20 m shuttle-run test.^44^

### Data analysis

For statistical analysis, the researchers opted to use the Statistical Package for the Social Sciences (SPSS) version 27. Mean values and standard deviations (s.d.) were used to describe and present the data. Because of the nature of the data and because the variables were not consistent to normality, a non-parametric Mann–Whitney *U-*test was used for establishing the significance of the gender differences on major theoretical variables of the study. Relationships of anthropometric and physiological characteristics with physical fitness characteristics of school learners were assessed using the Spearman’s rho correlation coefficient.

### Ethical considerations

The study protocol was approved by the research ethics committee of the hosting university and an ethical clearance certificate was issued as permission for the study to be conducted (ref. no. REC-270710-028-RA Level 01). Researchers also sought permission to conduct the study from the research ethics committees of the Department of Health as well as the Department of Education in the Eastern Cape province of South Africa. After receiving all the necessary ethical clearance letters, the researchers started data collection process. The scope and nature of the study were explained to the selected learners as well as their parents. The study involved learners aged 9–14 years and are regarded as minors which required parental consent to be obtained. Confidentiality and anonymity were maintained as guided by the study protocol.

## Results

From the sampled participants, the majority (*n* = 519; 59.66%) were girls and 351 (40.34%) were boys. In Table 1, the findings present the anthropometric and physiological features of the study sample. The sample had a mean age of 11.04 ± 1.50 years, with girls having significantly lower mean age (*M* = 10.91; s.d. = 1.48 years) as compared to that of boys (*M* = 11.24; s.d. = 1.51 years) (*p* = 0.002). The mean weight was 39.29 ± 10.34 kilograms. The mean height of the learners was 144.06 ± 10.94 cm. In gender differences on height, the findings revealed that there is no significant difference (*p* = 0.788) between girls (*M* = 143.91; s.d. = 11.08 cm) and boys (*M* = 144.28; s.d. = 10.40 cm). Boys had a significantly lower WC (*M* = 75.69; s.d. = 8.41 cm) and presented a lesser waist hip ratio (*M* = 1.21 ± 0.16 cm) than the girls (*M* = 79.08 ± 10.50 cm and 1.23 ± 0.30 cm, respectively). In addition, girls had significantly higher mean levels for gluteal (*M* = 65.02; s.d. = 9.11 cm), triceps (14.15 ± 6.09 mm) and subscapular (10.47 ± 6.54 mm) than that for boys. The results reveal a mean fat mass percentage of 20.04% (s.d. = 7.32%) with an overall mean fat mass of 8.34 kilograms (s.d. = 4.96) and a mean fat-free mass of 30.95 ± 6.51 kilograms. It is of paramount importance to note that, as compared to boys, girls had significantly higher mean levels for all these variables (all *p* ≤ 0.0001).

**TABLE 1:** Anthropometric and physiological features for the sampled participants.

Theoretical variables	Combined (*n* = 870)	Boys (*n* = 351)	Girls (*n* = 519)	Mann–Whitney *U Z*-test statistic	*p-*value
	*M* ± s.d.	*M* ± s.d.	*M* ± s.d.		
Age (years)	11.04 ± 1.50	11.24 ± 1.51	10.91 ± 1.48	−3.169	0.002*
Weight (kg)	39.29 ± 10.34	37.81 ± 8.53	40.29 ± 11.30	−2.735	0.006*
Height (cm)	144.06 ± 10.94	144.28 ± 10.40	143.91 ± 11.08	−0.269	0.788
BMI (kg/m^2^)	18.80 ± 4.11	18.04 ± 2.98	19.32 ± 4.65	−3.904	< 0.0001*
WC (cm)	77.72 ± 9.85	75.69 ± 8.41	79.08 ± 10.50	−4.953	< 0.0001*
WHR (cm)	1.22 ± 0.26	1.21 ± 0.16	1.23 ± 0.30	−2.633	0.008*
Gluteal (cm)	64.41 ± 9.12	63.51 ± 9.07	65.02 ± 9.11	−3.107	0.002*
Triceps (mm)	12.60 ± 6.17	10.30 ± 5.54	14.15 ± 6.09	−11.028	< 0.0001*
Subscapular (mm)	9.08 ± 5.97	7.01 ± 4.25	10.47 ± 6.54	−11.643	< 0.0001*
FATp (%)	20.04 ± 7.32	16.80 ± 6.98	22.24 ± 6.72	−12.016	< 0.0001*
FAT mass (kg)	8.34 ± 4.96	6.70 ± 4.35	9.45 ± 5.04	−9.654	< 0.0001*
FAT-free mass (kg)	30.95 ± 6.51	31.10 ± 5.74	30.84 ± 6.98	−1.323	0.186

*M*, Mean; s.d., standard deviation; BMI, body mass index; FAT mass, body fat mass; FATp, body fat percentage; FAT-free ***mass***, body fat free mass; WC, waist circumference; WHR, waist hip ratio.

* , statistically significant differences at alpha = 0.05.

Table 2 presents the descriptive statistics of the physical fitness characteristics of the school learners. Concerning push-ups, the sampled participants had a mean of 18.15 ± 9.41 push-ups per minute. Boys had statistically significant higher mean of push-ups than girls (*p* = 0.046). In terms of sit-ups, boys also had a significantly higher mean (*p* ≤ 0.0001) of sit-ups per minute (*M* = 24.07; s.d. = 13.50 per min) as compared to that of girls (*M* = 18.10; s.d. = 12.49 per min). Similar results were also reported for maximal oxygen uptake (VO2Max), where boys had significantly higher mean (*p ≤* 0.0001) of 31.88 as compared to that of girls (*M* = 28.64; s.d. = 8.05). However, girls had a significantly higher mean for sit and reach as compared to the boys (*p ≤* 0.0001).

**TABLE 2:** Physical fitness characteristics of the sampled participants.

Theoretical variables	Combined (*n* = 870)	Boys (*n* = 351)	Girls (*n* = 519)	Mann–Whitney *U Z*-test statistic	*p*
	*M* ± s.d.	*M* ± s.d.	*M* ± s.d.		
Push-ups (per min)	18.15 ± 9.41	19.15 ± 10.29	17.50 ± 8.71	−1.991	0.046*
Sit-ups (per min)	20.50 ± 13.23	24.07 ± 13.50	18.10 ± 12.49	−7.409	< 0.0001*
Sit & reach (cm)	23.94 ± 7.73	22.45 ± 7.86	24.95 ± 7.49	−4.629	< 0.0001*
VO2Max	31.88 ± 10.23	36.68 ± 11.20	28.64 ± 8.05	−11.053	< 0.0001*

*M*, Mean; s.d., standard deviation; VO2Max, maximal oxygen uptake.

* , statistically significant differences at alpha = 0.05.

Table 3 presents the Spearman’s rho correlation coefficients for the relationship of anthropometric and physiological characteristics with the physical fitness characteristics of school learners. These findings reveal that age had no statistically significant relationship with push-ups, as well as sit and reach in both the combined group and the boys-only group. Statistically significant positive relationships were reported for age and sit-ups, as well as VO2Max for the combined group and the boys-only group. Age had significant weak and positive relationship with sit and reach (*r_s_* = 0.096), as well as VO2Max (*r_s_* = 0.163) in the girls-only group.

**TABLE 3:** Relationships of anthropometric and physiological characteristics with physical fitness characteristics of school learners.

Variables	Combined *P*				Boys				Girls			
	Push-ups	Sit-ups	Sit and reach	VO2Max	Push-ups	Sit-ups	Sit and reach	VO2Max	Push-ups	Sit-ups	Sit and reach	VO2Max
		
	*r_s_*	*r_s_*	*r_s_*	*r_s_*	*r_s_*	*r_s_*	*r_s_*	*r_s_*	*r_s_*	*r_s_*	*r_s_*	*r_s_*
Age (years)	−0.006	0.133**	0.017	0.215**	0.050	0.202**	−0.057	0.236**	−0.060	0.047	0.096*	0.163**
Weight (kg)	−0.148**	−0.048	0.101**	−0.055	−0.081	0.022	0.001	0.031	−0.184**	−0.049	0.139**	−0.057
Height (cm)	−0.140**	0.038	0.023	0.097**	−0.082	0.087	−0.071	0.156**	−0.183**	0.024	0.078	0.073
BMI (kg/m^2^)	−0.124**	−0.122**	0.132**	−0.179**	−0.073	−0.065	0.087	−0.116*	−0.143**	−0.105*	0.136**	−0.150**
WC (cm)	−0.129**	−0.075*	0.122**	−0.110**	−0.059	−0.040	0.033	−0.019	−0.152**	−0.028	0.134**	−0.070
WHR (cm)	0.018	0.028	0.083*	0.049	0.006	0.033	0.069	0.049	0.036	0.065	0.074	0.114**
Gluteal (cm)	−0.132**	−0.082*	0.021	−0.129**	−0.064	−0.052	−0.074	−0.036	−0.168**	−0.067	0.055	−0.137**
Triceps (mm)	−0.109**	−0.202**	0.076*	−0.297**	−0.111*	−0.134*	0.070	−0.209**	−0.067	−0.098*	−0.005	−0.151**
Subscapular (mm)	−0.151**	−0.225**	0.126**	−0.274**	−0.099	−0.053	0.029	−0.130*	−0.163**	−0.181**	0.109*	−0.154**
FATp (%)	−0.135**	−0.229**	0.110**	−0.305**	−0.113*	−0.112*	0.059	−0.196**	−0.108*	−0.137**	0.057	−0.155**
FAT mass (kg)	−0.157**	−0.182**	0.117**	−0.236**	−0.100	−0.056	0.038	−0.114*	−0.160**	−0.117**	0.092*	−0.126**
FAT-free mass (kg)	−0.122**	0.032	0.078*	0.054	−0.047	0.079	−0.021	0.111*	−0.178**	−0.005	0.150**	−0.008

BMI, body mass index; FAT mass, body fat mass; FATp, body fat percentage; FAT-free mass, body fat free mass; WC, waist circumference; WHR, waist–hip ratio.

* , significant correlation at alpha = 0.05 (1-tailed test).

** , significant correlation at alpha = 0.01(1-tailed test).

Combined, weight had no significant relationship with sit-ups and VO2Max. However, in this group, weight had a weak, negative and significant relationship with push-ups (*r_s_* = – 0.148), whilst sit and reach had a weak, positive and significant correlation (*r_s_* = 0.101) with weight. This was also a similar finding in the girls-only group. In terms of the boys-only group, no significant relationships existed between weight and the physical fitness characteristics.

In terms of height, there exist significant, weak and negative correlations with push-ups in the combined group (*r_s_* = – 0.140) and girls-only group (*r_s_* = – 0.183). Weak, positive and significant relationships were reported on height and VO2Max in the combined group (*r_s_* = 0.097) and in the boys-only group (*r_s_* = 0.156).

Body mass index had significant correlations with all the physical fitness characteristics in the combined and girls-only groups. Of these, BMI had weak and negative significant relationships with push-ups, sit-ups and VO2Max, whilst sit and reach had a weak and positive significant relationship with BMI. For the boys-only group, BMI only had a significant but weak and negative relationship with VO2Max (*r_s_* = – 0.116).

All physical fitness characteristics had a significant relationship with WC in the combined group. Thus, there were significant, weak and negative correlations between WC and push-ups (*r_s_* = – 0.129), sit-ups (*r_s_* = – 0.095), as well as VO2Max (*r_s_* = – 0.110). There were no statistically significant relationships between WC and all physical fitness characteristics in the boys-only group. Looking at the girls-only group, WC had a weak, negative and significant relationship with push-ups (*r_s_* = – 0.152), whilst sit and reach had a significant, weak but positive relationship (*r_s_* = 0.134) with WC.

In terms of WHR, findings reveal that there only exist weak, positive and significant relationships with sit and reach (*r_s_* = 0.083) in the combined group and VO2Max (*r_s_* = 0.114) in the girls-only group. There were no statistically significant relationships between WHR and all physical fitness characteristics in the boys-only group.

In the combined group, gluteal had no significant relationship with sit and reach. However, push-ups (*r_s_* = – 0.132), sit-ups (*r_s_* = – 0.082) and VO2Max (*r_s_* = – 0.129) had weak, negative and significant correlations with gluteal. There were no statistically significant relationships between gluteal and all physical fitness characteristics in the boys-only group. On the other hand, in the girls-only group, gluteal had weak, negative but significant relationships with push-ups (*r_s_* = – 0.168) and VO2Max (*r_s_* = – 0.137).

All the physical fitness characteristics had a significant relationship with triceps in the combined group. Thus, there were significant, weak and negative correlations between triceps and push-ups (*r_s_* = – 0.109), as well as sit-ups (*r_s_* = – 0.202). VO2Max had a somewhat moderate, negative and significant relationship with triceps (*r_s_* = – 0.297). On the contrary, triceps had a weak, positive and significant relationship with sit and reach (*r_s_* = 0.097). In the boys-only group, triceps had no significant relationship with sit and reach, whilst push-ups (*r_s_* = – 0.111), sit-ups (*r_s_* = – 0.134) and VO2Max (*r_s_* = – 0.209) had weak, negative and significant correlations with triceps. In terms of the relationships in the girls-only group, triceps only had significant but weak and negative correlations with sit-ups (*r_s_* = – 0.098) and VO2Max (*r_s_* = – 0.151).

Findings also reveal that all the physical fitness characteristics had a significant relationship with subscapular in the combined group. Thus, there were significant, weak and negative correlations between subscapular and push-ups (*r_s_* = – 0.151) as well as sit-ups (*r_s_* = – 0.225). VO2Max had a somewhat moderate negative and significant relationship with subscapular (*r_s_* = –0.274). Alternatively, subscapular had a weak, positive and significant relationship with sit and reach (*r_s_* = – 0.126). A similar pattern of the relationships between subscapular and physical fitness characteristics was also reported in the girls-only group. As for the boys-only group, subscapular only had a significant relationship with VO2Max (*r_s_* = – 0.130), which is regarded as weak and negative.

Body fat percentage had significant correlations with all the physical fitness characteristics in the combined group. Of these, FATp had weak and negative significant relationships with push-ups (*r_s_* = – 0.135) and sit-ups (*r_s_* = – 0.229). VO2Max (*r_s_* = – 0.305) had a somehow moderate, negative and significant relationship with FATp, whilst sit and reach had a weak and positive significant relationship with FATp (*r_s_* = 0.110). In the boys-only group, FATp had no significant relationship with sit and reach, whilst push-ups (*r_s_* = – 0.113), sit-ups (*r_s_* = – 0.112) and VO2Max (*r_s_* = – 0.196) had weak negative and significant correlations with FATp. Also, in the girls-only group, FATp had no significant relationship with sit and reach, whilst push-ups (*r_s_* = – 0.108), sit-ups (*r_s_* = – 0.137) and VO2Max (*r_s_* = – 0.155) had weak negative and significant correlations with FATp.

FAT mass had significant correlations with all the physical fitness characteristics in the combined group. Of these, FAT mass had weak and negative significant relationships with push-ups (*r_s_* = – 0.157), sit-ups (*r_s_* = – 0.182), as well as VO2Max (*r_s_* = – 0.236). On the other hand, sit and reach had a weak and positive significant relationship with FAT mass (*r_s_* = 0.117). A similar pattern of the relationships between FAT mass and physical fitness characteristics was also reported in the girls-only group. As for the boys-only group, FAT mass only had a significant relationship with VO2Max (*r_s_* = – 0.114), which is regarded as weak and negative.

In terms of FAT-free mass, there exists no significant relationship with sit-ups as well as VO2Max. However, push-ups (*r_s_* = – 0.122) as well as sit and reach (*r_s_* = 0.098) had weak significant correlations with FAT-free mass. A similar pattern of the relationships between FAT-free mass and physical fitness characteristics was also reported in the girls-only group. As for the boys-only group, FAT-free mass only had a significant relationship with VO2Max (*r_s_* = – 0.114), which is regarded as weak and negative.

## Discussion

This current research study analysed how body composition relates to physical fitness amongst learners between 9 and 14 years of age. Findings revealed that BMI had significant negative correlations with three of the four physical fitness activities in girls (i.e. push-ups, sit-ups and VO2Max). In addition, girls had a significantly higher BMI than boys. Thus, as compared to boys, there is high prevalence of obesity or overweight amongst girls. This finding of higher BMI amongst the girls as compared to boys is supported by similar findings from a study conducted amongst Ugandan children.^29^ Paramount to the finding is that boys or girls with more BMI did fewer push-ups and sit-ups as compared to those with lesser BMI. This is supported by other research findings, which reported a negative association between body fat, higher body mass and physical fitness.^19,20,23,45^ Thus, learners with high BMI struggle to do strenuous, vigorous exercises such as push-ups, sit and reach, shuttle run and sit-ups.^23^ The results of the current study have shown that physical fitness performance between boys and girls can be understood based on the differences in gender which relate to body composition. As opposed to girls, boys have greater bone density, greater muscle mass and less body fat as compared to girls of the same age group.^46,47^ In support of this, it has been revealed in this study that excessive fatness negatively affects physical performance activities.

This study’s findings support studies from the developed countries. Thus, in developed countries, BMI and body fat have negative relationships with activities such as sit-ups and push-ups.^48^ In this current study, such relationships were found for sit-ups, push-ups and VO2Max across all groups. Thus, participants with a high BMI or body fat were performing worse in all the physical fitness activities in relation to time as compared with those with low BMI. Evidence from previous studies proves that learners who have high BMI are slower in shuttle run activities.^24,25^ In developed countries, these findings are because of the fact that BMI is regarded as an indicator of over-nourishment.^5,7,49,50^ This might not be the case in the South African context, as well as other developing countries, because there is no such thing as over-nourishment. In addition, in developed countries, BMI is a sign of fatness, whilst in middle-income countries such as South Africa it indicates higher muscle mass.^5,49^ Although there are contradictions on how BMI is viewed in developed countries as compared to middle and low-income countries, high BMI remains an indicator of physical inactivity. In contrast with the expectations in developed countries,^51^ this study has established that school learners (especially girls) with a higher BMI were able to do more sit and reach reps, which are a measure of flexibility. On the other hand, some studies reported that high BMI causes flexibility to decrease, which impacts negatively on physical performance.^52^ These positive correlations can be explained by the fact that physical fitness can not be defined through the structures of body composition alone but through other factors as well.

Some studies have reported that speed and acceleration capacity in VO2Max are affected by high BMI.^53,54^ Thus, for high physical activity performance there is a need for sufficient calories and oxygen, which is a challenge when there is high body fat, as it lowers cardiovascular endurance.^55^ Therefore, the relationship between body composition and physical performance is negative, as evidenced from the BMI and shuttle run across all groups. Thus, most of the learners with high body fat were finding it difficult to breathe and finish the screening test. Learners with low fat or rather FAT-free performed better in exercises because of superior power and strength in the muscle.^26^ Thus, these learners are reported to perform well in sporting activities such as the shuttle run.^24^ The current study also supports these findings. It should be noted that high body fat mass and high BMI have an impact on health-related physical fitness, which includes cardiorespiratory fitness, speed and power of movement.^56^ The results of this research allude to the fact that girls have low physical fitness; as a result, they also performed worse in physical activities.^5,7,57^ High BMI causes agility, strength and flexibility to decrease as well as cause loss of energy, which impacts negatively on physical performances.^52^

## Conclusion

The findings of the study revealed that, generally, there are negative relationships between body compositions and physical fitness characteristics. It has been revealed by the current study that physically, boys are stronger than girls, but on the other hand, girls are more flexible than boys. The study also revealed several significant and negative relationships between body composition measurements with physical fitness characteristics, which were stronger in girls than in boys. Furthermore, results of the study argue that there is a need for policymakers to design interventions and actions specifically suitable to the needs of predominantly rural school learners, especially in girls. Relevant stakeholders should implement targeted theory-based, contextually appropriate interventions^58,59^ which are aimed at promoting the increase of physical activities in rural-based learners mainly in the Eastern Cape province of South Africa.^60^ This would assist health professionals and also ensure that physical fitness amongst the learners in general improves.
